# Differentially accessible, single copy sequences form contiguous domains along metaphase chromosomes that are conserved among multiple tissues

**DOI:** 10.1186/s13039-021-00567-w

**Published:** 2021-10-20

**Authors:** Seana L. Hill, Peter K. Rogan, Yi Xuan Wang, Joan H. M. Knoll

**Affiliations:** 1 Department of Pathology & Laboratory Medicine, Schulich School of Medicine & Dentistry, University of Western Ontario, London, Canada; 2 Departments of Biochemistry and Oncology, Schulich School of Medicine & Dentistry, University of Western Ontario, London, Canada; 3 Cytognomix Inc., London, ON Canada

**Keywords:** Metaphase homologous chromosomes, Differential chromatin accessibility, Chromosome condensation, Epigenetics, Cytogenomics, Topologically associated domains (TADS), Tissue/cell types

## Abstract

**Background:**

During mitosis, chromatin engages in a dynamic cycle of condensation and decondensation. Condensation into distinct units to ensure high fidelity segregation is followed by rapid and reproducible decondensation to produce functional daughter cells. Factors contributing to the reproducibility of chromatin structure between cell generations are not well understood. We investigated local metaphase chromosome condensation along mitotic chromosomes within genomic intervals showing differential accessibility (DA) between homologs. DA was originally identified using short sequence-defined single copy (sc) DNA probes of < 5 kb in length by fluorescence in situ hybridization (scFISH) in peripheral lymphocytes. These structural differences between metaphase homologs are non-random, stable, and heritable epigenetic marks which have led to the proposed function of DA as a marker of chromatin memory. Here, we characterize the organization of DA intervals into chromosomal domains by identifying multiple DA loci in close proximity to each other and examine the conservation of DA between tissues.

**Results:**

We evaluated multiple adjacent scFISH probes at 6 different DA loci from chromosomal regions 2p23, 3p24, 12p12, 15q22, 15q24 and 20q13 within peripheral blood T-lymphocytes. DA was organized within domains that extend beyond the defined boundaries of individual scFISH probes. Based on hybridizations of 2 to 4 scFISH probes per domain, domains ranged in length from 16.0 kb to 129.6 kb. Transcriptionally inert chromosomal DA regions in T-lymphocytes also demonstrated conservation of DA in bone marrow and fibroblast cells.

**Conclusions:**

We identified novel chromosomal regions with allelic differences in metaphase chromosome accessibility and demonstrated that these accessibility differences appear to be aggregated into contiguous domains extending beyond individual scFISH probes. These domains are encompassed by previously established topologically associated domain (TAD) boundaries. DA appears to be a conserved feature of human metaphase chromosomes across different stages of lymphocyte differentiation and germ cell origin, consistent with its proposed role in maintenance of intergenerational cellular chromosome memory.

**Supplementary Information:**

The online version contains supplementary material available at 10.1186/s13039-021-00567-w.

## Background

The nucleotide sequence, associated chemical modifications, and proteins that package DNA in the nucleus determine the 3-dimensional architecture of chromosomes, both spatially and temporally [1]. The structural and functional organization of chromatin regulates differential gene expression programs essential for processes such as cell growth, division, differentiation and survival [1–3]. Alternating cycles of chromatin condensation and relaxation are interwoven amongst these programs producing the dynamic chromosome organization observed throughout the cell cycle. A high degree of condensation is necessary to ensure high fidelity segregation during cell division, however a more relaxed chromatin organization is needed for proper genome access by regulatory and transcriptional machinery to ensure normal cell function in interphase [3–5]. Despite constant changes in function and morphology within the cell cycle and during differentiation, new generations of cells are able to accurately re-establish cell (or functional) programming consistent with that of parent cells [6, 7]. The understanding of this mechanism remains incomplete. Epigenetic memory has been suggested as one mechanism to regenerate the same genome and epigenome organization in cell progeny [1, 8]. Identification and characterization of mechanisms of mitotic memory and bookmarking are ongoing with both tissue dependent and independent mechanisms proposed [9–13].

We have identified non-random, stable differences in condensation between homologous metaphase chromosome alleles (termed differential accessibility or DA) using fluorescence in situ hybridization with short single-copy (sc) sequence DNA probes (scFISH) [14–17]. DA is a manifestation of differences in chromatin supercoiling between metaphase homologs, which can be abrogated with an inhibitor of topoisomerase IIα [15]. DA has been observed in ~ 10% of scFISH probes developed and corresponding to single copy sequences within clinically relevant regions in the human genome [14, 16–18]. DA targets can include genic regions, exons and introns or intergenic sequences. Previous characterization of DA loci on human metaphase chromosomes has been performed with phytohemagglutinin (PHA)-stimulated lymphocytes [14, 15]. The role DA plays in the global condensation of chromosomes, transgenerational mitotic memory, or other aspects of nuclear organization remains unknown.

The transgenerational dynamics of chromosome condensation and relaxation must be consistent regardless of cell origin or genomic sequence. As a first step towards addressing these constraints, we examine genomic distribution of DA using linked sets of scFISH probes to define lengths of contiguous DA intervals. We also assessed whether DA was present at the same chromosomal loci among tissues at distinct somatic developmental stages and embryological origins (lymphocyte, bone marrow, and fibroblast cells). Characterizing the domain organization of DA in the genome and investigating DA among different cell types in which DA is found should provide clues into the role, if any, of local sequence compaction during metaphase chromosome condensation.

## Results

### Differential hybridization patterns for single copy (sc) probes confirmed on normal human metaphase chromosomes by scFISH

The genome distributions of DA intervals and their extent were addressed by FISH hybridization of multiple sc probes (1459–3553 bp) to 7 different chromosomal targets across 5 autosomes. Table 1 indicates scFISH probes used in this study to assess chromatin accessibility. It includes 19 probes, 18 scFISH probes with DA developed in this study and a previously developed 1p36 control probe with equivalent accessibility [EA] [14, 17], their chromosomal locations, and genome coordinates.

**Table 1:** Aggregated sc FISH probes and characteristics

Chromosome location	Domain Name [total length in bp]	Probe Name	Probe Genomic Coordinates [GRCh37/hg19]	Genomic Position*
1p36	N/A	3.3_1p36	chr1:1,171,789–1,175,143	Intergenic
2p23.1	XDH [25,454]	*XDH*_tel9263	chr2:31,545,815–31,547,924	Intergenic
		*XDH*_tel2386	chr2:31,551,816–31,554,801	Intergenic
		***XDH_*IVS30-IVS27**	chr2:31,568,769–31,571,269	*XDH*
3p24.3	HMGB1P5 [39,731]	***ZNF385D*_cen640649**	chr3:22,433,351–22,436,333	Intergenic
		*ZNF385D*_cen678130	chr3:22,470,832–22,473,082	Intergenic
12p12.3	FGF6 [16,048]	***FGF6*_tel4492**	chr12:4,537,157–4,538,816	Intergenic
		*FGF6_*IVS2	chr12:4,549,776–4,553,205	*FGF6*
15q21.1	N/A	*DUOX1*_IVS1-IVS3^a^	chr15: 45,422,890–45,424,597	*DUOX1*
15q22.2	TPM1 [16,034]	*TPM1*_IVS5-IVS8	chr15:63,353,573–63,355,980	*TPM1*
		***TPM1*_IVS8**	chr15:63,357,346–63,360,645	*TPM1*
		*TPM1*_tel3200	chr15:63,367,314–63,369,607	Intergenic
15q24.1	COX5A [109,970]	***SCAMP2_*IVS7-IVS4**	chr15:75,142,349–75,145,350	*SCAMP2*
		*SCAMP2_*IVS1	chr15:75,161,783–75,163,308	*SCAMP2*
		*COX5A_*tel20181	chr15:75,250,595–75,252,319	Intergenic
20q13.3	HMGB1P1 [129,583]	*RBM38_*tel25076	chr20:56,009,465–56,011,485	Intergenic
		***CTCFL*_cen34302**	chr20:56,033,167–56,036,719	Intergenic
		*PCK1*_cen13065	chr20:56,119,569–56,123,101	Intergenic
		*PCK1_*cen209*-*IVS6	chr20:56,135,957–56,139,048	*PCK1*

bp = base pair

^*^Genomic position refers to the target sequence hybridized by sc FISH probe. Intergenic refers to a sc probe target between genes or outside of a gene. A gene name indicates that the sc probe target is within that given gene including exons and introns

**Bolded probe names** indicate the anchor probe (initial DA probe identified) from which neighboring sc probes were developed,

^a^used in tissue conservation study not domain analysis

**Fig. 1:**
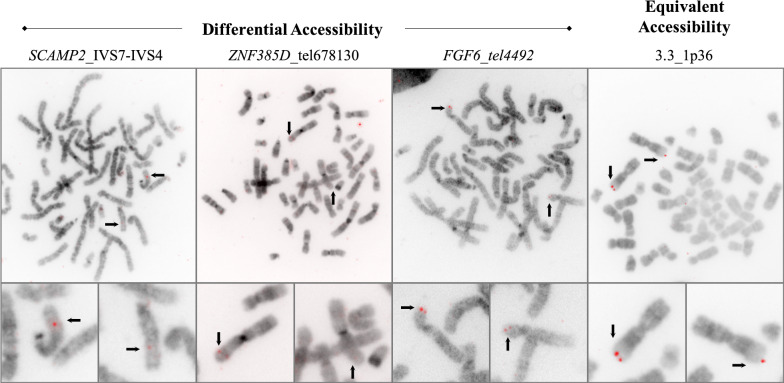
Regions of differential accessibility between human metaphase homologs detected by single copy probe FISH. T-lymphocyte metaphase cells hybridized with scFISH probes from *SCAMP2_*IVS7-IVS4 (3.002 kb; 15q24.1), *ZNF385D*_cen678130 (2.251 kb, 3p24.3) and *FGF6_*tel4492 (1.66 kb, 12p12.3) [top row, left to right] show differential hybridization intensity between homologs. Homolog with lowest probe fluorescence intensity and reduced accessibility is on the right. Arrows indicate expected probe target location on each homolog. Control scFISH probe 3.3_1p36 (3.354 kb; 1p36) shows similar fluorescence intensities (or equivalent accessibility [EA]) between homologous targets. Chromosomes were counterstained with DAPI. Probes were labelled with digoxigenin-11-dUTP and detected with Cy3-digoxin antibody. Cells were imaged using Metasystems Axioimager Z.2 epifluorescence microscope system with Metafer4 (V3.8.12) and Isis package (V5.3) imaging software. Images are presented in inverted gray scale

Chromosomal targets for the 6 anchor probes (bolded sequences) were selected from early human gene mapping studies, predating the publication of the full human genome sequence, in which published FISH images of metaphase chromosomes exhibited differences in hybridization intensities between homologs. We hypothesized that these differences in hybridization intensities might be related to DA (see Methods). In this regard, the selection of loci in this study purposefully differs from probes developed for our prior scFISH studies [16, 17, 19–21], which enriched for diagnostic EA probes present in expressed genes and in clinically relevant chromosomal regions. The initial DA sc probe developed for the chromosomal region targeted in each gene mapping publication [22–26] is bolded in Table 1. Figure 1 shows examples of hybridized metaphase cells with differences in probe fluorescence intensity between homologs for 3 DA probes: *SCAMP2_*IVS7-IVS4 (15q24.1), *ZNF385D*_cen678130 (3p24.3), *FGF6_*tel4492 (12p12.3) (first three panels). Below each metaphase cell panel, enlarged hybridized homolog pair images show DA. The homolog with less intense probe hybridization signal results from the chromosomal target being more condensed and less accessible for hybridization. A metaphase cell hybridized with a sc probe exhibiting equivalent accessibility (ie. EA) to both homologs: 3.3_1p36 (1p36.3) is also shown (4th panel). The 1p36 sc probe served as a control probe for EA and was described previously [14].

**Fig. 2:**
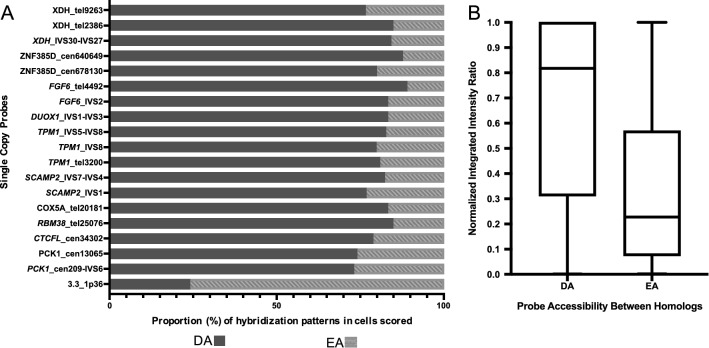
Quantitative properties of newly developed single copy probes. **A** Proportion of metaphase cells hybridized with single copy (sc) probes that exhibit different probe fluorescence intensities between homologs (differential accessibility [DA]; dark grey) and similar hybridization intensities (equivalent accessibility [EA]; medium grey). Each row represents unique sc probe data derived from combined metaphase FISH results of lymphocyte samples from two individuals. Probe names are listed in the left margin. For each of the first 18 probes, the majority of cells show DA hybridization pattern (73–89%) with a minor portion showing EA. The last row, control probe 3.3_1p36, shows that the opposite pattern. Ie. The majority of cells had an EA hybridization pattern (76%) with a minor portion showing DA. Significance between DA and EA probes was demonstrated using a two-tailed binomial test with normal approximation (α= 0.05). **B** Box and whisker plots of normalized integrated fluorescence intensity ratios between homologs. Intensity differences were quantified by GVF for 25 cells per probe. Five DA sc probes (*XDH_*IVS30-IVS27, *ZNF385D*_cen678130, *DUOX1_*IVS1-IVS3, *TPM1*_tel3200, *PCK1_*cen209*-*IVS6) demonstrated a large difference in median hybridization intensities between homologs relative to the sc EA probe (3.3_1p36). A significant difference was determined between the median integrated intensity ratio of DA (median intensity ratio = 0.82) and EA regions (median intensity ratio = 0.23) using a Mann–Whitney U test

Cytogenetic samples, prepared from PHA-stimulated peripheral blood from 23 different individuals, were used in this analysis to confirm chromosome location and determine hybridization pattern (DA or EA). ScFISH probe hybridized metaphase cells were initially analyzed qualitatively. For DA probes, a significantly greater proportion of cells demonstrated different probe hybridization intensities between homologs (73–89% of cells), in contrast to the control EA probe 3.3_1p36 that showed a greater proportion of cells with similar probe hybridization intensities between homologs (76% of cells) (Fig. 2A; Additional file 1: Table S1). This is consistent with our previous findings [14, 17]. Loci were determined to show differential accessibility using a two-tailed binomial test with normal approximation. The same test was used to determine equivalent accessibility of probe 3.3_1p36. A two proportion Z-test (α = 0.05) demonstrated no significant difference between the fraction of DA cells scored for the 23 different individual samples that established the accessibility pattern for 17 of the 18 DA probes. The *SCAMP2_*IVS7-IVS4 probe was an exception, in that while it also showed DA in both samples (ie. > 2/3 of cells, 75% [49/65 cells] vs 92% [44/48 cells]), the proportion of cells with DA differed between samples (*p* = 0.02). There was also no significant statistical difference in the fraction of cells with EA between individual samples hybridized with EA probe 3.3_1p36. Chromosomal accessibility differences between homologs are stable between unrelated individuals.

### Quantification of DA confirms qualitative DA classifications of new sc probes

Hybridizations on metaphase homologs from a subset of newly developed DA probes (*XDH_*IVS30-IVS27, *ZNF385D*_cen678130, *DUOX1_*IVS1-IVS3, *TPM1*_tel3200, *PCK1_*cen209*-*IVS6) and the control EA probe (3.3_1p36) were quantified using gradient vector flow (GVF) analysis [14, 27] to validate the qualitative analysis of DA and examine the extent of variation in hybridization intensity between homologs. GVF quantified fluorescence intensity of each homolog probe hybridization in each metaphase image. The difference in probe fluorescence between homologs was calculated as a normalized integrated intensity ratio between homologs in each metaphase cell. For each sc probe, the target pair of chromosome homologs in 25 cells were analyzed (DA, n = 125 diploid cells; EA, n = 25 diploid cells). A significant difference (*p* < 0.0001) was determined between the median intensity ratio of DA (0.82) and EA (0.23) probe targets using a Mann–Whitney non-parametric test (Fig. 2B). The interquartile range for DA regions is 0.31–1.00 whereas that of the single EA region is 0.07–0.57. This trend was consistent with previous published characterization of different DA probes and multiple EA regions [14].

### Relationship between open chromatin marks in interphase with mitotic accessibility characteristic of DA

Known open chromatin marks -DNase 1 hypersensitivity (DNase I HS), Formaldehyde Assisted Isolation of Regulatory Elements (FAIRE), and histone modifications (H3K4me, H3K9ac, H3K27ac, and H3K4me2) in lymphoblastoid cell line GM12878 were compared at the same genomic locations defined by the scFISH probes exhibiting DA and EA in lymphocytes during metaphase. The data reported are integrated intensity values from ENCODE data [28] reflecting chromatin accessibility during interphase (Additional file 2: Table S2). Overall, the mean integrated intensities of these 6 open chromatin marks in the newly identified DA regions (n = 17 of 18) were lower than those in equivalent accessibility (EA) sc intervals (n = 59 EA intervals, previously characterized in [14]; Additional file 3: Fig. S1A). The results, with the exception of one new DA probe, *SCAMP2_*IVS1, were consistent with the trend reported previously with a different set of DA probes [14]. The *SCAMP2_*IVS1 DA interval showed pronounced enrichment of open chromatin marks (2–75 fold difference with a mean ~ 18.4 fold increase), relative to the other DA loci in this study and those previously reported [14]. Open chromatin mark data for *SCAMP2_*IVS1 were excluded from the statistical analysis to prevent biased weighting of the intensity contributed by this probe sequence (Additional file 3: Fig. S1B, C).

### Aggregation of adjacent sc probes identifies DA domain organization in human metaphase homologs

**Fig. 3:**
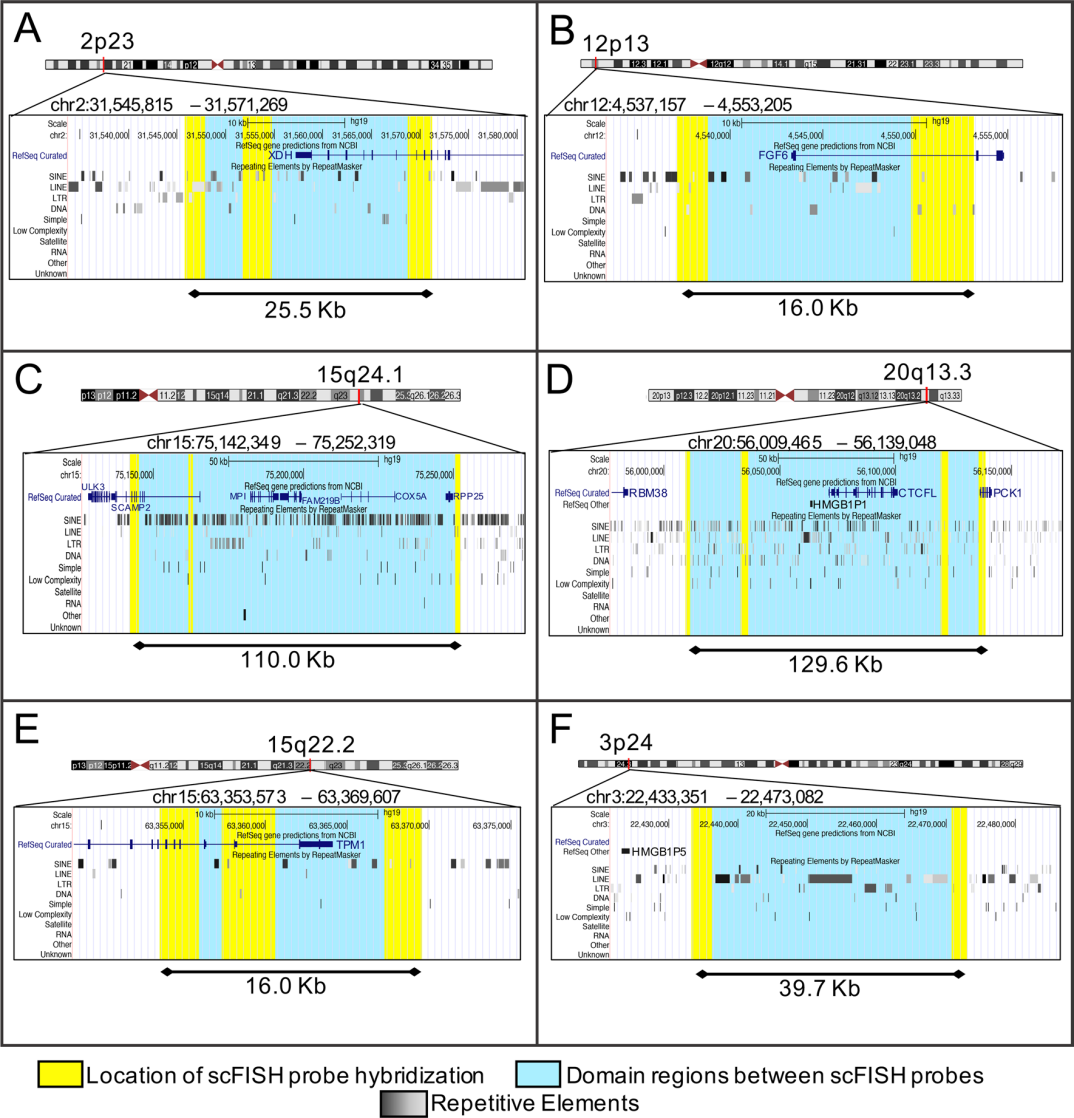
Genomic maps of 6 DA domains identified by neighbouring scFISH probes concordant for DA. Each map magnifies the genomic region of the DA domain from the chromosome band (red) highlighted on the chromosome ideogram. Created within the UCSC browser [40] using the GRCh37/hg19 human genome assembly, each domain is represented by turquoise and yellow bars. Yellow bars indicate the specific locations of the hybridized scFISH probes in each domain. The left margin names each track. Genomic coordinates [hg19] are provided followed by curated RefSeq genes (dark blue) and a variety of different repetitive sequences. The repeating elements (RepeatMasker) are presented in greyscale. Decreasing intensity of grey to white corresponds to increasing divergence between DNA sequences within the same family. ScFISH probes are located within regions that either have no repeating elements or divergent repeating elements (> 20% sequence divergence). Hybridized sc probes are listed left to right in panel descriptions: **A** XDH domain spans 25.455 kb and includes scFISH probes: *XDH*_tel9263, *XDH*_tel2386, *XDH_*IVS30-IVS27. **B** FGF6 domain spans 16.048 kb and includes scFISH probes: *FGF6*_tel4492, *FGF6_*IVS2. **C** COX5A domain spans 109.970 kb and includes scFISH probes: *SCAMP2_*IVS7-IVS4, *SCAMP2_*IVS1, *COX5A_*tel20181. **D** HMGB1P1 domain spans 129.583 kb and includes scFISH probes: *RBM38_*tel25076, *CTCFL*_cen34302, *PCK1*_cen13065, *PCK1_*cen209*-*IVS6. **E** TPM1 domain spans 16.034 kb and includes scFISH probes: *TPM1*_IVS5-IVS8, *TPM1*_IVS8, *TPM1*_tel3200. **F** HMGB1P5 domain spans 39.731 kb and includes scFISH probes: *ZNF385D*_cen640649, *ZNF385D*_cen678130

To determine the extent of DA in these targeted regions, we evaluated metaphase epigenotypes of sc probes within the same chromosomal regions. Neighbouring single copy intervals, in the vicinity of DA anchor probes were scored for metaphase accessibility. When adjacent probes were scored as concordant for DA, they constituted a chromosomal DA domain. Domains are named according to the gene localized in the legacy FISH gene mapping publication from which sc probes were derived: XDH (chr 2p23), HMGB1P5 (chr 3p24), FGF6 (chr 12p13), TPM1 (chr 15q22), COX5A (chr 15q25) and HMGB1P1 (chr 20q13) (Table 1, Fig. 3). The boundaries of each domain are demarcated by the smallest and largest genome coordinates of the two maximally separated DA probes. A given domain is inferred to be contiguous between DA probes. The content of the sc probe targets can comprise genic introns, exons and intergenic regions within 6 different chromosomal regions on 5 chromosomes.

The integrated intensities of open chromatin marks (DNase I HS and FAIRE) of individual scFISH probes of each DA domain in lymphoblastoid cell line GM12878 were evaluated and compared with those values across the full domain (Additional file 2: Table S2). For both DNase I HS (Domain µ = 1.42, scFISH μ = 2.01; p = 0.18) and FAIRE (Domain µ = 5.28 scFISH μ = 5.62; p = 0.49), there were no significant differences between the integrated intensity of open chromatin marks per base pair between DA scFISH probes and the domain they defined (using an unpaired t-test with Welch correction).

The DA domains range in size from 16.034 kb to 129.583 kb and consist of 2 to 4 DA sc targets identified within each domain (Table 1; Fig. 3A–F). Three of the DA domains span short genomic intervals. The TPM1 domain (chr 15q22.1, Fig. 3E) spans 16.034 kb and is defined by 3 DA probes which have a combined total length of 8.0 kb. Two of the probes span introns 5 to 8 of *TPM1* and the third probe is intergenic and distal to *TPM1*. The FGF6 domain (chr 12p12; Fig. 3B) covers a genomic distance of 16.048 kb and is defined by 2 DA probes totaling 5.1 kb target length. One probe in this domain targets intron 2 of *FGF6* and the other is intergenic and distal to *FGF6*. The XDH domain is defined by 3 DA probes spanning 25.454 kb of chromosome 2p23.1 (Fig. 3A) with a probe target length of 7.6 kb. Two of the DA probes in this domain are intergenic and distal to *XDH* and the other spans introns 27 to 30 of *XDH*. The close proximity of the multiple sc probes within each of these domains indicates that differential accessibility between homologs likely extends beyond the defined genomic coordinates of the single probes contained within aggregate chromosomal intervals.

The other 3 domains show DA over greater genomic lengths. The HMGB1P5 domain (chr 3p24.3, Fig. 3F) is entirely intergenic and defined by 2 DA probes spanning a 39.731 kb genomic distance. The probe targets combined total 5.2 kb in length. The two largest domains, COX5A (Fig. 3C; 15q24.1) and HMGB1P1 (Fig. 3D; 20q13.3) span 109.97 kb and 129.583 kb, respectively. The COX5A domain is defined by 3 DA probe regions distal to *COX5A*, 2 within *SCAMP2* (IVS4-IVS7; IVS1) and one in an intergenic region with a total target length of 6.3 kb. HMGB1P1 is defined by 4 DA probes, 3 from intergenic sequences adjacent to *RBM38*, *CTCFL* and *PCK1* and one from within *PCK1* (5’ end to IVS6) with a 12.2 kb target length.

Demonstration of 3 and 4 DA intervals within COX5A and HMGB1P5 domains, respectively, (without interspersion of EA intervals) supports the possibility that long range, possibly contiguous DA regions may be common in the genome. It was not possible to delimit the full extent or contiguous nature of these domains, as the sc probes themselves did not cover the entire genomic span of the inferred domains. The COX5A domain (Fig. 3C) contained an 87.4 kb region without sc probe coverage between *SCAMP2_*IVS1 and *COX5A_*tel20181; and the HMGB1P1 domain (Fig. 3D) exhibited an 82.9 kb gap between the *CTCFL*_cen34302 and *PCK1*_cen13065 probes, and smaller gaps of < 25 kb between the other probes.

### DA is conserved among different cell types

**Fig. 4:**
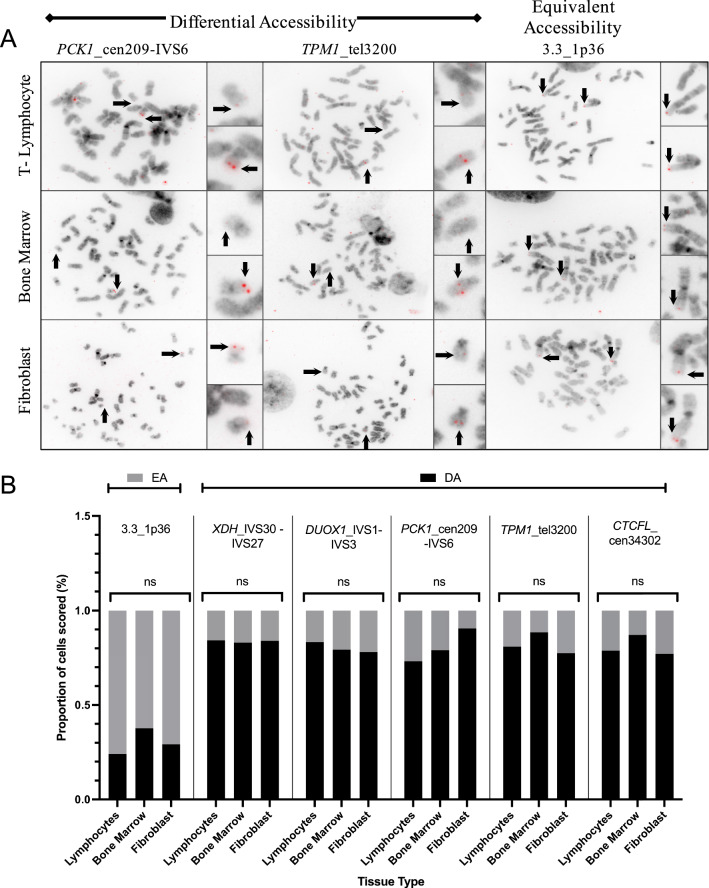
Chromatin accessibility patterns between metaphase homologs are conserved between different cell types. **A** Human metaphase cells from T-lymphocyte (top row), bone marrow (center row) and fibroblast (bottom row) cells hybridized with scFISH probes *PCK1*_cen209-IVS6 (chr 20q13.3, left column), *TPM1*_tel3200 (chr 15q22, center column), and 3.3_1p36 (right column). Hybridized homologs are indicated with arrows on the metaphase cells and enlarged homologs. The differential hybridization intensity observed across all tissues at the *PCK1*_cen209-IVS6 (left) and *TPM1*_tel3200 (center) loci are characteristic of differential accessibility (DA). Equivalent hybridized probe intensities observed at locus 3.3_1p36, characteristic of equivalent accessibility (EA) are also conserved between all tissues. Probe 3.3_1p36 serves as a control as an EA locus. Chromosomes were counterstained with DAPI. Probes were labelled with digoxigenin-11-dUTP and detected with Cy3-digoxin antibody. Cells were imaged using Metasystems Axioimager Z.2 epifluorescence microscope system with Metafer4 (V3.8.12) and Isis package (V5.3) imaging software. Images presented in inverted gray scale. **B** Proportion of cells scored with DA (black) within lymphocytes, bone marrow cells, and fibroblasts are not significantly different from each other when hybridized with sc probes for *XDH*_IVS30-IVS27, *DUOX1*_IVS1-IVS3, *PCK1*_cen209-IVS6, *TPM1*_tel3200 and *CTCFL*_cen34302. Three of these DA probe targets (*XDH*, *DUOX1*, *PCK1*) are within genes and the other two (*TPM1* and *CTCFL*) are within intergenic regions. Proportion of cells scored with EA (light grey) across tissue types did not significantly differ from each other when hybridized with EA probe, 3.3_1p36. Across the tissues examined for each DA or EA region, the accessibility between metaphase homologs remained the same. Sample size differs between each tissue and each probe (Additional file 4: Table S3). Significant differences were calculated using a Kruskal Wallis test (α = 0.05) comparing between tissues and proportion of cells scored as DA and EA

Five scFISH probe loci that exhibit DA in peripheral blood PHA-stimulated lymphocytes were also evaluated in bone marrow and fibroblast tissues, and the observed DA patterns in metaphase cells were consistent among these tissues and different individuals (Fig. 4A). These characteristic differences in probe fluorescence between metaphase homologs were observed with genic probes *XDH*_IVS30-IVS27, *PCK1*_cen209-IVS6 (Fig. 4A, left), *DUOX1*_IVS1-IVS3 and intergenic probes *TPM1*_tel3200 (Fig. 4A, center), and *CTCFL*_cen34302. Individual scFISH probe analysis was generally performed on two samples for each tissue type (ie. 6 hybridizations per probe) with 25 or more metaphase cells scored per sample. The exceptions (due to sample mitotic index limitations) were for probe *TPM1*_tel3200 which was analyzed on a single fibroblast sample and a single bone marrow sample; and probes *DUOX1*_IVS1-IVS3 and *XDH*_IVS30-IVS27 which were each analyzed on one fibroblast sample.

Open chromatin marks were analyzed for sequences of sc probes in dermal fibroblast cell lines GM03348 (DNase I HS) and NHDF-Ad (histone modifications: H3K4me, H3K9ac, H3K27ac, and H3K4me2). The regions corresponded to each DA interval in this study and to previously reported EA intervals [14]. In fibroblasts, EA intervals showed a higher mean integrated intensity of all open chromatin marks analyzed in interphase relative to DA intervals, consistent with our results for lymphocytes both in this study (Additional file 2: Table S2) and previous DA characterizations [14]. Further, no significant difference was found between the mean integrated intensities of DA regions in lymphocytes and fibroblasts for all marks (DNase I HS *p* = 0.75, H3K4me *p* = 0.75, H3K27ac *p* = 0.66, and H3K4me2 *p* = 0.095) except one (H3K9ac p = 0.03) (Additional file 2: Table S2). FAIRE was not analyzed as data from a normal dermal fibroblast line was not available in the UNC FAIRE data set.

Using a two proportion Z-test (α = 0.05), the fraction of cells with DA for different probes was also similar between individuals for different samples (n = 6 bone marrow, 2 fibroblast, 10 PHA-stimulated peripheral blood lymphocyte). The only exception was for probe *CTCFL*_cen34302, in which 2 bone marrow samples had significant differences between the proportion of cells with DA (α = 0.004; 98% [44/45 cells] vs 76% [38/49 cells] of cells). The DA pattern for *CTCFL*_cen34302 was not different between the two lymphocyte samples or the two fibroblast samples analyzed. DA was indistinguishable between T-lymphocytes, bone marrow, and fibroblasts at each locus (p > 0.99 based on the Kruskal–Wallis test) (Fig. 4B). The proportion of cells scored as DA for each DA locus across tissues were significant using a normally distributed two-tailed binomial test (α = 0.05) (Additional file 4: Table S3). The chromosome 1p36 EA control probe also showed EA across all samples and cell types, with no difference between individuals (Fig. 4B, Additional file 4: Table S3).

DA conservation at the same loci in T-lymphocytes and bone marrow cells suggests DA is present and maintained in B-lymphocytes, as well as progenitor cells at various stages of differentiation. Fibroblasts yielded similar results suggesting DA, once established, is retained in tissues derived from both ectoderm and mesoderm germ layers.

## Discussion

This study confirms and extends candidate DA regions identified by visually comparing FISH intensity differences between metaphase homolog images in legacy gene mapping publications [22–26, 29]. Sc probes developed from these regions were assessed for DA or EA, confirming that biased hybridization intensity differences in these studies were likely the result of DA, rather than from technical aspects of hybridization to recombinant DNA-based probes. This conclusion was reinforced by probes from neighboring genomic intervals that also exhibited DA.

The chromosomal distribution and extent of adjacent differentially accessible intervals between homologs in metaphase—whether isolated or clustered in domains—had not been investigated until the present study. In interphase, differences in the epigenetic structures of an 8.16 mb region of chromosome 19 homologs showed non-random differences in accessibility and volume, whose structures were highly variable between cells [30]. We describe 6 different DA domains, XDH, FGF6, COX5A, TPM1, HMGB1P1 and HMGB1P1, of varying lengths on different chromosomes. Domains in homologous metaphase chromosomes appear to be organized as contiguous sc intervals showing differential accessibility. Furthermore, these domains appear to be conserved along mitotic chromosomes of different germline origins and hematopoietic differentiation states.

The TPM1, XDH and FGF6 domains consist of tightly clustered DA regions. By contrast, the HMGB1P5, COX5A, and HMGB1P1 domains, contain larger gaps between the DA regions confirmed using scFISH (although in some regions, probe development was constrained by the minimum lengths and densities of the single copy intervals). This raises the intriguing possibility that DA occurs more often within neighbouring single copy regions than we have previously described. Based on our previous work which identified DA in ~ 10% of scFISH probes, it seems plausible that expansion of these domains by linking adjacent short sc intervals likely increases the overall proportion of mitotic chromatin that may be subject to DA [14].

None of the DA loci defining individual domains adjoin one another. The shortest distance between DA loci is ~ 1.4 kb, and the largest ~ 87.3 kb. The major constraint in designing single copy FISH probes was related to the intrinsic distribution of repetitive elements within these regions. Sequences containing repetitive elements with divergent sequences < 20% from consensus family members were excluded from probe design to avoid nonspecific cross-hybridization across the genome [14, 16, 17, 31]. It was also not possible to identify EA sequences flanking DA intervals, despite intensive efforts to delimit boundaries of DA domains by selecting sc intervals of increasing distances from the anchor DA probe. Previously published work from our laboratory has demonstrated that ~ 90% of single-copy probes from regions derived from clinically relevant genes/genomic regions exhibit EA [14]. For this reason, it is likely that the boundaries of the domains described here will eventually be circumscribed by adjoining sc intervals displaying EA.

Recent models of metaphase chromatin organization have suggested that oligomeric, nucleosomal, associated protein-DNA, and spacer complexes can be structured as multi-layered intercalated plates or as stacked thin-layered solenoids [32, 33]. It may be possible to reconcile differences between these models by incorporating regional differences in catenation of chromatin [15]. Our proposed model of DA suggests a difference in the numbers of topoisomerase-induced supercoils, i.e. winding number, between homologs without changes in loop frequency or helical pitch [15]. Structurally, this aligns well with the proposed multi-layer plate metaphase folding model that is in equilibrium between a condensed and relaxed state [32–34]. This particular model also aligns best with chromosome banding and band splitting along the length of the chromosome [32, 33]. However, these models cannot address why the compaction states of some allelic segments of metaphase homologs would be consistently different (ie. exhibit DA). Also, these models do not account for differences in extended DA domains in homologous chromosomes or their conservation among tissues with different origins.

The presence of DA domains in metaphase, rather than isolated DA intervals, is consistent with the proposal that these features of metaphase homologs may be correlated with or be precursors to topologically associated domains (TADs) re-established during interphase. TADs have been suggested to form coherent structural units of (primarily) cis-interacting genomic sequences in interphase chromatin [35, 36]. TADs facilitate interactions with regulatory elements and their gene targets within the defined boundaries of chromatin scaffolds. These interphase organizations are almost certainly eliminated during mitosis to allow condensation of chromatin [4, 5], including the loss of transcription factors important in establishing compartmentalization (e.g. CTCF) in interphase; therefore, understanding the mechanisms responsible for re-establishing these interactions in daughter cells is of considerable interest.

**Fig. 5:**
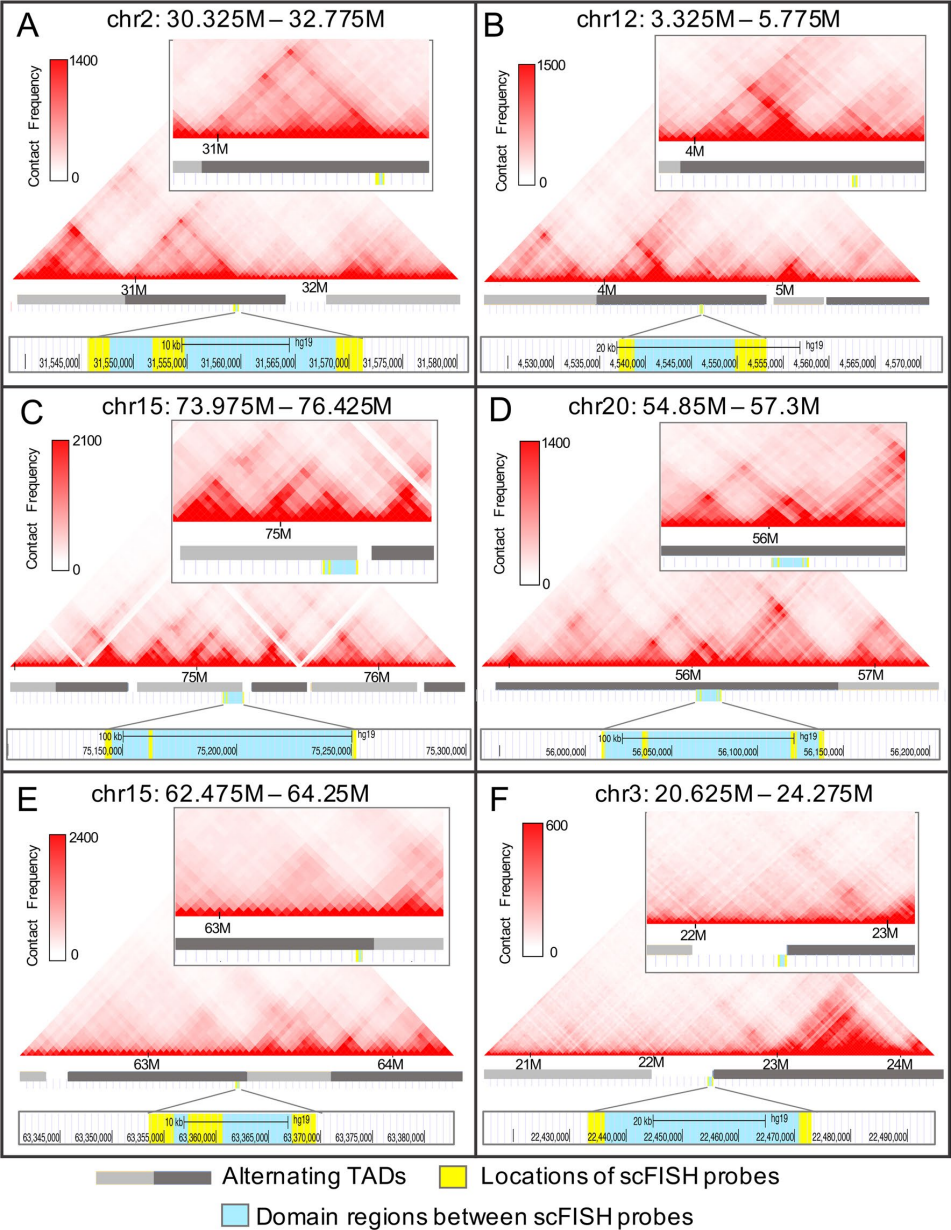
Localization of DA domains in metaphase homologs relative to topologically associated domains (TADs) in interphase. The heat map (generated by 3D Genome Browser [38]) shows interaction frequencies between chromatin within each domain (yellow-turquoise areas) and surrounding chromatin in UCSC Genome Browser image (GRCh37/hg19). Each heatmap spans the chromosome coordinates indicated above. Alternating dark grey and light grey bars represent separate TADs assigned by the 3D Genome Browser. The scale in top left measures the intensity of contact frequency as the normalized number of contacts between 2 points. The frequency of intra-TAD interactions within and around each domain can be assessed by areas of frequent contact shown as bright red triangular signals occurring above the domain. Red intensity increases with higher frequency of interactions. The long-range interactions (white, pale red) > million bp in panels A-F have been covered to accommodate the insets magnifying intra-TAD interactions within each domain. **A** XDH domain localizes within a single TAD (dark grey). The XDH domain is within a section of genome with multiple intra-TAD interactions observed by multiple areas of high contact frequency at varying distances. **B** FGF6 domain localizes within a single TAD (dark grey). **C** COX5A domain is at one end of a single TAD (light grey). **D** HMGB1P1 domain is in the middle of a single TAD (dark grey) with frequent interactions occurring with loci in proximity with the domain. **E** TPM1 domain localizes within a single TAD (dark grey) approaching the boundary with the adjacent TAD. There are some intra-TAD interactions with sequences close to the domain but compared to other DA domains, they are fewer and less frequent. **F** The HMGB1P5 domain is the only domain not within a TAD. The proximity of the domain to the boundary of the TAD (dark grey) can be clearly seen

In this study, to assess correspondence between loci of DA in metaphase and proximate interphase TAD structures, chromatin confirmation capture information from Hi-C analysis in lymphoblast cell-line GM12878 [37] was visualized for DA domains and sc probe intervals using the 3D Genome Browser [38]. Five of the six DA domains defined in metaphase chromosomes (XDH, FGF6, COX5A, TPM1, HMGB1P1) each correspond to an interphase region contained within a single TAD (Fig. 5A–E). Domains XDH (Fig. 5A), FGF6 (Fig. 5B), and HMGB1P1 (Fig. 5D) correspond to sequences in the middle of their respective TADs, while COX5A (Fig. 5C) and TPM1 (Fig. 5E) domains approach a TAD boundary. The other DA domain, HMGB1P5, occurs between adjacent TADs proximate to one of the TAD boundaries (Fig. 5F). The extent of intra-chromosomal contacts or compartments within the corresponding DA domains (and flanking regions) is indicated by heat maps showing relative contact frequencies (Fig. 5A–F). The insets highlight the overlap of DA domains with areas of frequent localized short-range intra-chromatin interactions and looping which suggest compartments within the larger TAD structure [35, 37].

The topology of interphase chromosomes based on 5C studies indicates numerous interactions between neighboring sequences within the same TAD. Interaction between neighboring DA segments has not been documented in this or previous studies. Nevertheless, the mitotic epigenotypes of these individual segments appears to be consistent with extensive condensation or catenation levels in these regions on the same homolog. Catenation differences between homologs revealed by DA may be associated with differences in chromatin folding and their association with gene expression and regulation during the subsequent interphase [6, 9, 35].The preponderance of metaphase DA domains corresponding to sequences each occurring within a self-contained TAD in interphase, is consistent with DA serving as a structural link that conserves large-scale chromatin organization between mitotic and interphase chromosomes. These findings motivate a thorough genome-wide analysis of the alignment of DA domains with Hi-C chromatin conformation data underlying TAD structures. This would clarify whether the epigenetic relationships noted here between metaphase and interphase chromatin organizations are generalizable.

FISH signal intensities in these DA domains were consistent with previous comparisons of reported DA and EA regions [14]. Epigenetic open chromatin marks of the probes in this study were also consistent with our previous analyses of other DA probes. DA loci exhibit reduced characteristics of open chromatin (DNase 1 Hypersensitivity (DNase I HS), Formaldehyde Assisted Isolation of Regulatory Elements (FAIRE), and histone modifications H3K4me, H3K9ac, H3K27ac, and H3K4me2) compared to previously described loci with equivalent accessibility [14]. We also found mean integrated intensity values of DNase I, H3K9ac, H3K27ac, and H3K4me2 at DA loci were significantly lower, except for FAIRE and H3K4me where the differences were not significant. The *SCAMP2_*IVS1 genomic interval was an outlier in that it was highly enriched for these open chromatin marks, which likely reflects the proximity of this probe sequence to the *SCAMP2* promoter that is highly expressed in B-lymphocytes (https://gtexportal.org/home/gene/SCAMP2). Further, the integrated intensity per base pair of open chromatin marks analyzed for scFISH probe coverage of each domain was representative of the overall contiguous domain in lymphocytes.

Initially, DA loci and domain characterization were defined using peripheral T- lymphocytes. It is now apparent that DA at these loci is conserved in bone marrow and dermal fibroblasts as well. Three DA loci within genes and two in intergenic regions were identified in all tissues. The samples from all individuals showed DA for all probes tested, and the proportions of cells exhibiting DA with a specific probe were generally similar between samples from different individuals or tissue type (T-lymphocytes, bone marrow, fibroblasts). Analysis of (largely) interphase open chromatin marks in fibroblasts were analogous to those seen at these loci in lymphocytes (i.e., lower mean integrated intensities in new DA regions relative to EA regions). Differences between open chromatin marks in lymphocytes and fibroblasts of these new DA regions was largely unremarkable and revealed marginally significant differences only involving H3K4 acetylation. This suggests that the same epigenetic characteristics used to define DA regions during interphase in lymphocytes could also be used for fibroblasts. The mitotic cells in bone marrow would include both B lymphocytes and other progenitor cells at different stages of differentiation. That the same DA domains occur during multiple stages of hematopoiesis and from different germ layers (mesoderm: lymphocytes and ectoderm: fibroblast) suggests that establishment of DA may be an innate property of mitotic chromosome condensation. If DA is a stable chromatin mark throughout development, then its presence in both mesoderm and ectoderm derived cells would indicate its establishment in early embryogenesis. The establishment of DA structures early in mitosis distinguishes homologs and could represent a transgenerational mechanism that preserves sister chromatid identity after cell division. Such a mechanism would be consistent with our previous findings that demonstrate conservation of DA between inherited or derivative chromosomes [14].

## Methods

### Single copy probe design, development of probes for fluorescence in situ hybridization (FISH)

Methods for scFISH have been described previously [14–17, 31]. The overall process for FISH probe development involved precise definition of each single copy (sc) interval by specific human genome coordinates and range in length from ~ 1.4 to 4 kilobases (kb). The sequence of each sc interval was amplified from human genomic DNA with polymerase chain reactions (PCR) optimized for long products, followed by the gel purification of amplicons, and labelling by nick translation with a modified nucleotide (digoxigenin-11-dUTP) prior to performing hybridization to metaphase chromosomes. Following hybridization, probes were detected with a fluorescence labeled antibody against digoxigenin on metaphase chromosomes stained with 4’,6-diamidino-2-phenylindole (DAPI). Cells were imaged using a Metasystems computer assisted epifluorescence microscope system.

Sc DNA probes were comprised of either unique DNA sequences or highly divergent repetitive sequences (> 20%) that behave as unique sequence targets during chromosomal hybridization [14–17, 31]. Sc genomic intervals were excluded if they were present in copy number variants with ≥ 1% population frequency [14] and were observed in independent microarray datasets, including Ontario Population Genomics Platforms (n = 873 individuals of European ancestry; minimum 25 probes per CNV; Database of Genomic Variants), and Healthy sample set (n =  ~ 400 individuals; minimum 35 probes per CNV, Affymetrix), which were used to identify common CNVs with ChAS (Chromosome Analysis Suite) software analysis of ThermoFisher (formerly Affymetrix) CytoScan HD arrays (Additional file 5: Table S4).

### Oligonucleotide primer design and sc amplicon production

Primer pairs for each selected sc interval were designed using Primer-BLAST [39]. Sc intervals were identified using RepeatMasker (University of California Santa Cruz (UCSC) Genome Browser). The DNA sequence (GRCh37/hg19) for the full sc interval, obtained from the UCSC Genome Browser [40] was the PCR template used to generate all primer pair options. Generally, 15–20 primer pairs were designed for each sc interval. The maximum size of the PCR product was limited by the length of the sc interval in base pairs (bp) and the minimum length was 200–500 bp less than the maximum. The selected primer melting temperature (Tm) range was 58.0—65.0 °C, with an optimal Tm of 62.0 °C. The maximum Tm difference between a pair of primers was limited to 2 °C. Primer pair specificity was verified using the “RefSeq representation genome” database for alignment with the human genome by BLAST® (Basic Local Alignment Search Tool) [39] as well as separate assessment by BLAT (GRCh37/hg19 and CHM13 [41]). The nucleotide coordinates of the primer pairs reported from the GRCh38/hg38 genome assembly were converted to GRCh37/hg19 coordinates using the UCSC genome browser. Optimal primer pairs minimized the self-complementarity of individual primers and the Tm difference between the pair. Primers in which the PCR product had unintended targets and generally those outside the 40–60% GC content range were avoided. Longer primers (> 25 bp) were preferred. Primer pairs were synthesized by Integrated DNA Technologies, Inc (Toronto, ON). Long PCR reaction conditions using hot start DNA polymerase Kappa HiFi (Promega Corporation) according to the manufacturer’s instructions were optimized for each sc interval using a gradient PCR thermocycler (Eppendorf vapo.protect™ Hamburg, Germany). Optimized PCR conditions were then used for scale-up of the target amplicons. The amplicons were gel purified and labelled by nick translation for use in fluorescence in situ hybridization [14, 17, 42]. The primer details and PCR optimization cycling parameters are provided in Additional file 6: Table S5.

### Cytogenetic preparations

Cytogenetic fixed cell preparations were obtained from phytohemagglutinin (PHA)-stimulated peripheral blood, bone marrow, and dermal fibroblast samples. The cytogenetic cell preparations were derived from de-identified residual cell pellets that remained after routine cytogenetic diagnostic procedures were completed at the London Health Sciences Center Clinical Cytogenetics Laboratory (University of Western Ontario Office of Research Ethics, CER approval #5453). Cytogenetically normal cell pellets were used for bone marrow samples. Cell pellets were produced following routine cytogenetic protocols for cell culture and harvest [14] and fixed with 3 parts methanol: 1 part glacial acetic acid (Carnoy’s fixative).

Fibroblast metaphase cells of normal adults were also prepared in the research laboratory by culturing dermal fibroblast cells stored in liquid nitrogen in our research laboratory cell bank [43]. Fibroblasts were cultured in T25 flasks at 37 °C/5% CO_2_ in DMEM – Dulbecco’s Modified Eagle Medium (Gibco #11,960–044) supplemented with 15% fetal bovine serum (Hyclone #SH30396.03) and 1% penicillin/streptomycin (Hyclone #SV30010). Cultures were grown until ~ 70% confluent, arrested in metaphase with colcemid (Gibco#15,212–012) and harvested [43]. Fibroblasts were treated with hypotonic solution at 37 °C (0.075 M KCl) and fixed with Carnoy’s fixative. Fixed cell preparations were placed on glass microscope slides and aged at room temperature (1–3 days) prior to performing scFISH.

### Sc probe selection for examining DA domains

All sc probes in Table 1 were developed in this study, with the exception of sc probe 3.3_1p36 [14, 17], which is a control probe showing EA (Additional file 7, Figure S2). For each domain, these consisted of anchor probes with confirmed DA as well as multiple scFISH probes linked in the genome to these anchor sc probes. The anchor probes were designed and produced from genomic regions corresponding to published legacy chromosomal localization studies of *XDH* [22], *HMGB1P5* and *HMGB1P1* [23], *FGF6* [24], *TPM1* [25], and *COX5A* [26]. These genes map to chromosome bands 2p23 (XDH), 3p24 (HMGB1P5), 12p13 (FGF6), 15q22 (TPM1), 15q25 (COX5A), and 20q13 (HMGB1P1). Legacy publications that mapped genes on human chromosomes by FISH were identified thru PubMed and journal searches. Many of these gene mapping studies were published prior to the initial assembly of the complete human genome sequence in 2001. The ‘gene mapping’ FISH probes [22–26, 29] generally consisted of recombinant DNA with long human genomic inserts that ranged in length from ~ 50 kb to several hundred kb, and in which the full genomic sequence was not known. We scrutinized the FISH images in these publications to identify potential differences in the fluorescence hybridization intensities of signals hybridizing to each chromosome homolog, which are characteristic of DA. Images that appeared to exhibit differential hybridization were further characterized in our laboratory by scFISH to determine whether the published intensity differences met our criteria for DA. The locations of the FISH probe genomic targets were determined using the probe specific gene mapping details, such as restriction enzyme mapping and partial gene sequencing in these or related publications, which were then used to computationally localize sc intervals in the current human genome assembly. Sc probes were developed from within the large genomic target regions using previously published methods [14–20]. If DA was determined to be present by scFISH, the sc probe then served as an anchor probe from which to develop neighboring probes. The neighboring probes were used to determine if DA extended beyond the anchor sequence and formed a larger DA domain.

All sc probes developed in this study, were hybridized to lymphocyte metaphase chromosomes to confirm the expected chromosomal band location and then scored for hybridization pattern (ie. DA or EA) as summarized below using our previously described methods [14, 15, 17]. Domains were named based on the HUGO-approved gene name in the corresponding legacy gene mapping publication from which the anchor probe was derived. Sc probes are named according to their location within or adjacent to the gene from which it was derived. In intergenic regions, probes are identified by the coding gene closest to the sc interval, with centromeric (cen) or telomeric (tel) indicating the position of the probe relative to that gene, and followed by the distance in nucleotides between the gene and interval. Probes localizing within genes are named with the gene and the interval of exons and introns spanned, guided by conventions stipulated by Human Genome Variation Society (HGVS) nomenclature.

### Scoring differential (DA) and equivalent accessibility (EA) of sc probe hybridization between metaphase homologous chromosomes—qualitative and quantitative

Evaluation of differences in the hybridized probe fluorescence intensity between homologs was performed as previously reported [14, 15]. Chromosome identification and scoring of the intensity of hybridized probe fluorescence signals (dim, medium, bright) was performed independently by a minimum of 2 analysts. A metaphase cell was considered to show differential accessibility (DA) if homologs were scored with different intensities (e.g. bright/medium, bright/dim, medium/dim, bright/nil). A cell was scored as equivalently accessible (EA) when homologs were scored with equivalent intensities (e.g. bright/bright, medium/medium). Any scores of dim/dim, nil/nil, or dim/nil were excluded. Cells with hybridized chromosomes involved in chromosome overlap at or near the location of probe hybridization were also excluded to rule out potential hybridization effects on the targets. Twenty-five or more cells were scored for most samples, and a minimum of 2 samples were evaluated per scFISH probe for probe validation. A two-tailed binominal test with normal approximation was used to determine if there was a significant difference between the proportion of DA cells compared to that of EA cells [14]. Additionally, a two proportion Z-test was used to test if the proportion of DA cells differed between samples. Both statistical tests were performed at α = 0.05.

Visual differences in hybridized probe fluorescence intensities between homologs within the same cell were quantified using the gradient vector flow algorithm (GVF) that we previously developed [14, 27]. GVF determines FISH probe boundaries for each chromosomal hybridization as a binary contour and integrates the probe fluorescence across the subset of pixels comprising each signal [27]. Integrated signal intensity for homologs 1 and 2 are defined as ${I1}\; {\text{and}}\; {I2}$, respectively. To determine differences between the signals of each homolog within a cell, a normalized intensity ratio was calculated:

$$ Intensity\,\, Ratio = \frac{|I1-I2|}{I1+I2}$$

Values close to 0 indicate homologs with EA, whereas values close to 1 are differences in signal intensity present in DA [14]. A bias in hybridization signal intensities between homologous regions was reported as statistically significant using a Mann–Whitney U test.

### Sc probe selection for investigating DA in different cell types

To avoid confounding factors such as differential tissue expression that could influence chromatin accessibility, sc probes were selected from within genes that had little to no expression (0.0–5.0 transcripts per million [TPM]) across all tissues of interest (lymphocytes/blasts, bone marrow, fibroblast). Expression data in TPM were downloaded from the Genotype-Tissue Expression (GTEx) [44] and Human Protein Atlas [45, 46] databases. GTEx expression data were from EBV transformed lymphocytes and fibroblasts with multiple samples representing each tissue. The mean and standard deviation across samples was computed with a homebrew Python script. The Human Protein Atlas data were derived from multiple bone marrow samples and obtained as mean expression values. A subset of sc probes that demonstrated DA in T-lymphocytes developed during this study were selected to assess whether DA at these loci was conserved in bone marrow cells and fibroblasts. DA intervals present within genes (intronic and exonic) as well as in intergenic intervals, were selected to establish DA across different tissues in both gene coding and noncoding intervals. The probes selected within genes were *XDH*_IVS30-IVS27, *PCK1*_cen209-IVS6, and *DUOX1*_IVS1-IVS3. Intergenic DA regions that were assumed to be transcriptionally inactive from UCSC genome browser annotations included *TPM1*_tel3200 and *CTCFL*_cen34302. *DUOX1*_IVS1-IVS3 sc probe (chr 15q23) genomic region was developed and validated after review of historical FISH images within a *SORD* gene mapping study [29].

### Sequence comparison of epigenetic open chromatin marks between single copy probe genomic intervals exhibiting DA or EA

Epigenetic features characteristic of open chromatin were analyzed following the same approach that we have previously reported for other EA and DA genomic intervals [14]. The open chromatin properties extracted from the Encyclopedia of DNA Elements (ENCODE) [28] that were compared with mitotic accessibility included: DNase I hypersensitivity (Duke, Dnase1 HS), Formaldehyde-Assisted Isolation of Regulatory Elements (FAIRE) (University of North Carolina, FAIRE seq) and histone marks H3K4me1, H3K9ac, H3K27ac, and H3K4me2 (Broad Institute, histone modifications). All open chromatin marks reported were derived from data collected from the Epstein-Barr virus (EBV) transformed lymphoblastoid cell line, GM12878, in which DA had previously been characterized [14], and untransformed dermal fibroblast lines: GM03348 (DNase I HS) and NHDF-Ad (H3K4me, H3K9ac, H3K27ac, and H3K4me2). All histone modification data were derived from ChIP-seq (chromatin immunoprecipitation assay with sequencing) signal intensities. The cumulative sum of signals for each open chromatin mark was determined for all sc intervals, and a mean integrated intensity was calculated for DA and EA groups individually. Box and Whisker plots of each mark for both DA and EA visualized these distributions. Unpaired t-tests with Welch correction were used to test for significant differences (α = 0.05) between the mean integrated intensity of each chromatin mark between DA and EA intervals in lymphocytes and fibroblasts as well as integrated intensity per base pair between full DA domains and scFISH domain coverage. The open chromatin marks for new DA probes developed in this investigation were compared to previously reported EA probe intervals [14]. Open chromatin mark data for *SCAMP2*_IVS2 were censored from the other DA interval data set prior to statistical testing between DA and EA loci. *SCAMP2_*IVS1 is within intron 1, a gene segment in which promoters have been identified [47, 48], which paired with the pronounced enrichment of open chromatin marks is consistent with *SCAMP2*_IVS1 localizing within the highly accessible *SCAMP2* promoter. This sequence is not representative of the predominantly intergenic locations (n = 10) that characterize the other DA probes; therefore, *SCAMP2*_IVS1 was excluded from the analysis of the above interphase chromatin features, in order to prevent biased weighting of the total integrated intensities by probe sequences.

### Higher order chromatin structures in DA domains

The organization of DA domain intervals with respect to higher-order chromatin structures, topologically associated domains (TADs), was analyzed using the public 3-D genome browser [38] with chromatin capture data (Hi-C) of lymphoblast cell line GM12878 [37]. Chromatin interaction frequency heatmaps were generated at a resolution of 25 kb spanning DA domain and sc probe locations (GRCh37/hg19) within the UCSC genome browser [40]. Correspondence of DA domains with TADs and other intra-TAD interactions were analyzed from scaled heat-map and genome browser outputs from the 3-D Genome Browser and UCSC Genome Browser, respectively [38, 40].

## Supplementary Information

**Additional file 1.** **Table S1**: ScFISH probes developed and validated to evaluate the extent of DA domains established with an anchor scFISH probe and conservation of DA between tissues.

**Additional file 2.** **Table S2**: (A) Integrated intensity values of open chromatin marks in each DA and EA interval from this study, (B) Integrated intensity values of open chromatin marks for each DA domain.

**Additional file 3.** **Figure S1**: Open chromatin marks at DA loci have lower mean integrated intensities compared to EA loci. A) Integrated intensity values of DA regions (red) were significantly lower than EA regions (black) of DNase I HS, H3K9ac, H3K27ac, and H3K4me2 using an unpaired t-test with Welch’s correction for unequal variances. No significant difference was found between the mean integrated intensity values of DA and EA regions for FAIRE and H3K4me (p>0.05). The 95% confidence intervals for the DA (n = 17, excluding SCAMP2_IVS1) and previously reported EA intervals [n=59, (14)] are shown. B) Distribution of integrated intensity data for each open chromatin mark in new DA intervals (n=18) and previously reported EA intervals (n=59). Data for each open chromatin mark (x-axis) are presented in a box and whisker plot with the limits of each whisker determined by Tukey. Center line of each box represents the median. Outliers are represented by dots beyond the limits of the whiskers of each box plot. A single outlier from the DA group identified in 5 of 6 open chromatin marks was derived from the same interval, SCAMP2_IVS1. C) Genomic map of COX5A domain demonstrates the difference in enrichment of open chromatin mark H3K27ac at the SCAMP2_IVS1 DA probe outlier, compared to the neighbouring DA probe loci SCAMP2_IVS7-IVS4 and COX5A_tel20181. H3K27ac (burgundy) is enriched at the SCAMP2_IVS1 locus relative to all other DA loci in this study and those previously reported. H3K27ac signal is ChIP-seq data from the GM12878 lymphoblast cell line (Broad Institute). UCSC genome browser annotations are indicated for GRCh37/hg19. RefSeq genes with isoforms are dark blue, sc probes with DA loci within chromosome region 15q24.1 are yellow. Turquoise indicates the domain defined by these probes.

**Additional file 4.** **Table S3**: DA and EA cell count for each probe and tissue type examined with results of significance testing between DA and EA proportions per probe and between individuals.

**Additional file 5.** **Table S4**: Frequency of copy number variants (CNVs) that overlap DA intervals used for scFISH probes.

**Additional file 6.** **Table S5**: Details for the production of all new ScFISH probes developed in this study including PCR cycling parameters.

**Additional file 7.** **Figure S2**: Genomic map of EA scFISH probe 3.3_1p36. The map expands the genomic EA region of 3.3_1p36 from the chromosome band (red) highlighted on the chromosome ideogram. Created within the UCSC browser (40) using the GRCh37/hg19 human genome assembly, the yellow bar indicates the specific location of the hybridized scFISH probe (chr1:1,171,789-1,175,143). The left margin names each track. Genomic coordinates [hg19] are provided followed by curated RefSeq genes (dark blue when present) and a variety of different repetitive sequences. An intergenic region, no genes are present in this genomic map. The repeating elements (RepeatMasker) are presented in greyscale. Decreasing intensity of grey to white corresponds to increasing divergence between DNA sequences within the same family. ScFISH probes are located within regions that either have no repeating elements or divergent repeating elements (greater than 20% sequence divergence).

## Data Availability

Supplementary data and materials are provided in: (i) Additional Table S1 – Sample DA Frequencies for sc probes to evaluate extent of DA domains established with anchor scFISH probe and conservation between tissues. (ii) Additional Table S2 – Total Integrated Intensities for Open Chromatin Marks for each probe in the study. (iii) Additional Table S3 – Tissue DA and EA cell frequency for each probe and tissue type with significance testing. (iv) Additional Table S4 – CNV information for DA intervals used for sc probes. (v) Additional Table S5—Primers for ScFISH probes Details and PCR Amplification Conditions. (vi) Additional Fig. S1- Statistical analysis and comparison of ENCODE open chromatin marks between DA and EA sc probe regions with and without outlier probes. (vii) Additional Fig. S2 – Genomic map of control EA scFISH probe 3.3_1p36. The following are web locations used with the probes listed in Table 1, Additional Table S5 and are included within the manuscript. (i) The Encyclopedia of DNA Elements (ENCODE; 10.1371/journal.pbio.1001046) integrated intensity data that support findings in this study are available in the UCSC genome browser (https://genome.ucsc.edu), the tracks used are DNase I hypersensitivity (Duke Dnase1 HS), Formaldehyde-Assisted Isolation of Regulatory Elements (FAIRE) (University of North Carolina FAIRE seq) and histone marks H3K4me1, H3K9ac, H3K27ac, and H3K4me2 (Broad Institute histone modifications). (ii) The HiC chromatin interaction data in our study are available from the 3D genome browser (10.1186/s13059-018-1519-9, http://3dgenome.fsm.northwestern.edu/view.php) using data from lymphoblast cell line GM12878. (iii) Expression data in TPM were downloaded from the Genotype-Tissue Expression (GTEx, https://gtexportal.org/home/, 10.1038/ng.2653) and Human Protein Atlas (https://www.proteinatlas.org) databases to determine sc probe intervals with little or no expression in tissues investigated for chromatin accessibility conservation. (iv) CHM13 chromosome assembly, http://genome.ucsc.edu/cgi-bin/hgTracks?genome=t2t-chm13-v1.0&hubUrl=http://t2t.gi.ucsc.edu/chm13/hub/hub.txt, https://www.biorxiv.org/content/10.1101/2021.05.26.445798v1

## Abbreviations

BLAST®: Basic Local Alignment Search Tool
BLAT: BLAST Like Alignment Tool
ChIP-seq: Chromatin Immunoprecipitation Assay with Sequencing
CNV: Copy Number Variation
*COX5A*: Cytochrome C Oxidase Subunit 5A
*CTCFL*: CCCTC-Binding Factor Like
DA: Differential Accessibility
DNase I HS: Deoxyribonuclease I Hypersensitivity
*DUOX1*: Dual Oxidase 1
EA: Equivalent Accessibility
EBV: Epstein-Barr Virus
ENCODE: Encyclopedia of DNA Elements
FAIRE: Formaldehyde-Assisted Isolation of Regulatory Elements
*FGF6*: Fibroblast Growth Factor 6
GTEx: Genotype-Tissue Expression database
GVF: Gradient Vector Flow
*HMGB1P1*: High Mobility Group Box 1 Pseudogene 1
*HMGB1P5*: High Mobility Group Box 1 Pseudogene 5
H3K4me: Histone 3 Lysine 4 Methylation
H3K4me2: Histone 3 Lysine 4 Dimethylation
H3K9ac: Histone 3 Lysine 9 Acetylation
H3K27ac: Histone 3 Lysine 27 Acetylation
sc: Single-copy
*PCK1*: Phosphoenolpyruvate Carboxykinase 1
PHA: Phytohemagglutinin
*RPM38*: RNA Binding Motif Protein 38
*SCAMP2*: Secretory Carrier Membrane Protein 2
TAD: Topologically Associated Domain
*TPM1*: Tropomyosin 1
*XDH*: Xanthine Dehydrogenase
*ZNF385D*: Zinc Finger Protein 385D

## References

[1] The 4D Nucleome Network, Dekker J, Belmont AS, Guttman M, Leshyk VO, Lis JT, et al. The 4D nucleome project. Nature. 2017;549(7671):219–26. 10.1038/nature23884 PMC5617335 28905911

[2] Nozaki T, Imai R, Tanbo M, Nagashima R, Tamura S, Tani T (2017). Dynamic organization of chromatin domains revealed by super-resolution live-cell imaging. Mol Cell.

[3] Yu S, Yang F, Shen WH (2016). Genome maintenance in the context of 4D chromatin condensation. Cell Mol Life Sci.

[4] Naumova N, Imakaev M, Fudenberg G, Zhan Y, Lajoie BR, Mirny LA (2013). Organization of the mitotic chromosome. Science.

[5] Gibcus JH, Samejima K, Goloborodko A, Samejima I, Naumova N, Nuebler J (2018). A pathway for mitotic chromosome formation. Science.

[6] Golloshi R, Sanders JT, McCord RP (2017). Genome organization during the cell cycle: unity in division. Wiley Interdiscip Rev Syst Biol Med..

[7] Martínez-Balbás MA, Dey A, Rabindran SK, Ozato K, Wu C (1995). Displacement of sequence-specific transcription factors from mitotic chromatin. Cell.

[8] Lodhi N, Ji Y, Tulin A (2016). Mitotic bookmarking: maintaining post-mitotic reprogramming of transcription reactivation. Curr Mol Biol Rep.

[9] Festuccia N, Gonzalez I, Owens N, Navarro P (2017). Mitotic bookmarking in development and stem cells. Development.

[10] Creyghton MP, Cheng AW, Welstead GG, Kooistra T, Carey BW, Steine EJ (2010). Histone H3K27ac separates active from poised enhancers and predicts developmental state. Proc Natl Acad Sci.

[11] Liu Y, Pelham-Webb B, Di Giammartino DC, Li J, Kim D, Kita K (2017). Widespread mitotic bookmarking by histone marks and transcription factors in pluripotent stem cells. Cell Rep.

[12] Bellec M, Radulescu O, Lagha M (2018). Remembering the past: Mitotic bookmarking in a developing embryo. Curr Opin Syst Biol.

[13] Oomen ME, Dekker J (2017). Epigenetic characteristics of the mitotic chromosome in 1D and 3D. Crit Rev Biochem Mol Biol.

[14] Khan WA, Rogan PK, Knoll JH. Localized, non-random differences in chromatin accessibility between homologous metaphase chromosomes. Mol Cytogenet [Internet]. 2014 Dec;7(1). Available from: http://molecularcytogenetics.biomedcentral.com/articles/10.1186/s13039-014-0070-y 10.1186/s13039-014-0070-y PMC4269072 25520753

[15] Khan WA, Rogan PK, Knoll JHM. Reversing chromatin accessibility differences that distinguish homologous mitotic metaphase chromosomes. Mol Cytogenet [Internet]. 2015 Dec;8(1). Available from: http://www.molecularcytogenetics.org/content/8/1/65 10.1186/s13039-015-0159-y PMC4535684 26273322

[16] Rogan PK, Cazcarro PM, Knoll JHM (2001). Sequence-based design of single-copy genomic DNA probes for fluorescence in situ hybridization. Genome Res.

[17] Knoll JHM, Rogan PK (2003). Sequence-Based, in situ detection of chromosomal abnormalities at high resolution. Am J Med Genet A.

[18] Khan WA, Knoll JH, Rogan PK (2011). Context-based FISH localization of genomic rearrangements within chromosome 15q11.2q13 duplicons. Mol Cytogenet.

[19] Mora JR, Knoll JHM, Rogan PK, Getts RC, Wilson GS (2006). Dendrimer FISH detection of single-copy intervals in acute promyelocytic leukemia. Mol Cell Probes.

[20] Knoll JHM, Rogan PK (2004). High resolution definition of chromosome abnormalities with probes designed from genome sequences. Discov Med.

[21] Rogan PK, Knoll JHM. High resolution detection of chromosome abnormalities with single copy fluorescence in situ hybridization. In: 2004 2nd IEEE International Symposium on Biomedical Imaging: Macro to Nano (IEEE Cat No 04EX821) [Internet]. Arlington, VA, USA: IEEE; 2004 [cited 2021 Aug 10]. p. 73–6. Available from: http://ieeexplore.ieee.org/document/1398477/

[22] Rytkönen EM, Halila R, Laan M, Saksela M, Kallioniemi OP, Palotie A (1995). The human gene for xanthine dehydrogenase (XDH) is localized on chromosome band 2q22. Cytogenet Cell Genet.

[23] Rogalla P, Borda Z, Meyer-Bolte K, Tran KH, Hauke S, Nimzyk R (1998). Mapping and molecular characterization of five HMG1-related DNA sequences. Cytogenet Genome Res.

[24] Chaffanet M, Baens M, Aerssens A, Schoenmakers E, Cassiman J-J, Marynen P (1995). Mapping of an ordered set of 14 cosmids to human chromosome 12p by two-color in situ hybridization. Cytogenet Genome Res.

[25] Eyre H, Akkari PA, Wilton SD, Callen DC, Baker E, Laing NG (1995). Assignment of the human skeletal muscle &alpha;-tropomyosin gene (TPM1) to band 15q22 by fluorescence in situ hybridization. Cytogenet Genome Res.

[26] Hofmann S, Lichtner P, Schuffenhauer S, Gerbitz K-D, Meitinger T (1998). Assignment^1^ of the human genes coding for cytochrome c oxidase subunits Va (COX5A), VIc (COX6C) and VIIc (COX7C) to chromosome bands 15q25, 8q22→q23 and 5q14 and of three pseudogenes (COX5AP1, COX6CP1, COX7CP1) to 14q22, 16p12 and 13q14→q21 by FISH and radiation hybrid mapping. Cytogenet Genome Res.

[27] Arachchige AS, Samarabandu J, Knoll J, Khan W, Rogan P. An image processing algorithm for accurate extraction of the centerline from human metaphase chromosomes. In: 2010 IEEE International Conference on Image Processing. 2010. p. 3613–6.

[28] The ENCODE Project Consortium. A User’s Guide to the Encyclopedia of DNA Elements (ENCODE). Becker PB, editor. PLoS Biol. 2011;9(4):e1001046. 10.1371/journal.pbio.1001046 PMC3079585 21526222

[29] Lee FK, Cheung MC, Chung S (1994). The Human Sorbitol Dehydrogenase Gene: cDNA Cloning, Sequence Determination, and Mapping by Fluorescence in Situ Hybridization. Genomics.

[30] Nir G, Farabella I, Pérez Estrada C, Ebeling CG, Beliveau BJ, Sasaki HM, et al. Walking along chromosomes with super-resolution imaging, contact maps, and integrative modeling. Copenhaver GP, editor. PLOS Genet. 2018;14(12):e1007872. 10.1371/journal.pgen.1007872 PMC6324821 30586358

[31] Dorman SN, Shirley BC, Knoll JHM, Rogan PK (2013). Expanding probe repertoire and improving reproducibility in human genomic hybridization. Nucleic Acids Res.

[32] Chicano A, Crosas E, Otón J, Melero R, Engel BD, Daban J. Frozen‐hydrated chromatin from metaphase chromosomes has an interdigitated multilayer structure. EMBO J [Internet]. 2019 Apr [cited 2021 Aug 10];38(7). Available from: 10.15252/embj.201899769 10.15252/embj.201899769 PMC6443200 30609992

[33] Daban J-R (2015). Stacked thin layers of metaphase chromatin explain the geometry of chromosome rearrangements and banding. Sci Rep.

[34] Wu C, McGeehan JE, Travers A (2016). A metastable structure for the compact 30-nm chromatin fibre. FEBS Lett.

[35] Dixon JR, Gorkin DU, Ren B (2016). Chromatin domains: the unit of chromosome organization. Mol Cell.

[36] Dixon JR, Selvaraj S, Yue F, Kim A, Li Y, Shen Y (2012). Topological domains in mammalian genomes identified by analysis of chromatin interactions. Nature.

[37] Rao SSP, Huntley MH, Durand NC, Stamenova EK, Bochkov ID, Robinson JT (2014). A 3D map of the human genome at kilobase resolution reveals principles of chromatin looping. Cell.

[38] Wang Y, Song F, Zhang B, Zhang L, Xu J, Kuang D, et al. The 3D Genome Browser: a web-based browser for visualizing 3D genome organization and long-range chromatin interactions. Genome Biol [Internet]. 2018 Dec [cited 2019 Apr 19];19(1). Available from: https://genomebiology.biomedcentral.com/articles/10.1186/s13059-018-1519-9 10.1186/s13059-018-1519-9 PMC6172833 30286773

[39] Ye J, Coulouris G, Zaretskaya I, Cutcutache I, Rozen S, Madden TL. Primer-BLAST: A tool to design target-specific primers for polymerase chain reaction. BMC Bioinformatics [Internet]. 2012 Dec [cited 2019 May 5];13(1). Available from: https://bmcbioinformatics.biomedcentral.com/articles/10.1186/1471-2105-13-134 10.1186/1471-2105-13-134 PMC3412702 22708584

[40] UCSC Genome Browser Home [Internet]. 2019 [cited 2019 Nov 6]. Available from: https://genome.ucsc.edu/index.html

[41] Nurk S, Koren S, Rhie A, Rautiainen M, Bzikadze AV, Mikheenko A, et al. The complete sequence of a human genome [Internet]. Genomics; 2021 May [cited 2021 Jun 23]. Available from: http://biorxiv.org/lookup/doi/10.1101/2021.05.26.445798

[42] Knoll JHM, Lichter P, Bakdounes K, Eltoum I-EA. In Situ Hybridization and Detection Using Nonisotopic Probes. Curr Protoc Mol Biol. 2007 Jul;79(1):14.7.1–14.7.17. 10.1002/0471142727.mb1407s79 18265392

[43] Priest JH. Chapter 4 General Cell Culture Principles and Fibroblast Culture. In: Barch MJ, Knutsen Turid, Spurbeck JL, Association of Genetic Technologists., editors. The AGT cytogenetics laboratory manual. 3rd ed. Philadelphia: Lippincott-Raven Publishers; 1997.

[44] Lonsdale J, Thomas J, Salvatore M, Phillips R, Lo E, Shad S (2013). The genotype-tissue expression (GTEx) project. Nat Genet.

[45] Uhlén M, Fagerberg L, Hallström BM, Lindskog C, Oksvold P, Mardinoglu A (2015). Tissue-based map of the human proteome. Science.

[46] E-MTAB-2836 < Browse < ArrayExpress < EMBL-EBI [Internet]. 2019 [cited 2019 May 10]. Available from: https://www.ebi.ac.uk/arrayexpress/experiments/E-MTAB-2836/

[47] Lu R, Rogan PK (2018). Transcription factor binding site clusters identify target genes with similar tissue-wide expression and buffer against mutations. F1000Research..

[48] Eddy J, Maizels N (2008). Conserved elements with potential to form polymorphic G-quadruplex structures in the first intron of human genes. Nucleic Acids Res.

