# Supplementary figures and images for: Differentially accessible, single copy sequences form contiguous domains along metaphase chromosomes that are conserved among multiple tissues
Source: Mol Cytogenet. 2021 Oct 20;14:49. doi: 10.1186/s13039-021-00567-w (PMC8527651; doi:10.1186/s13039-021-00567-w)

Figure S1

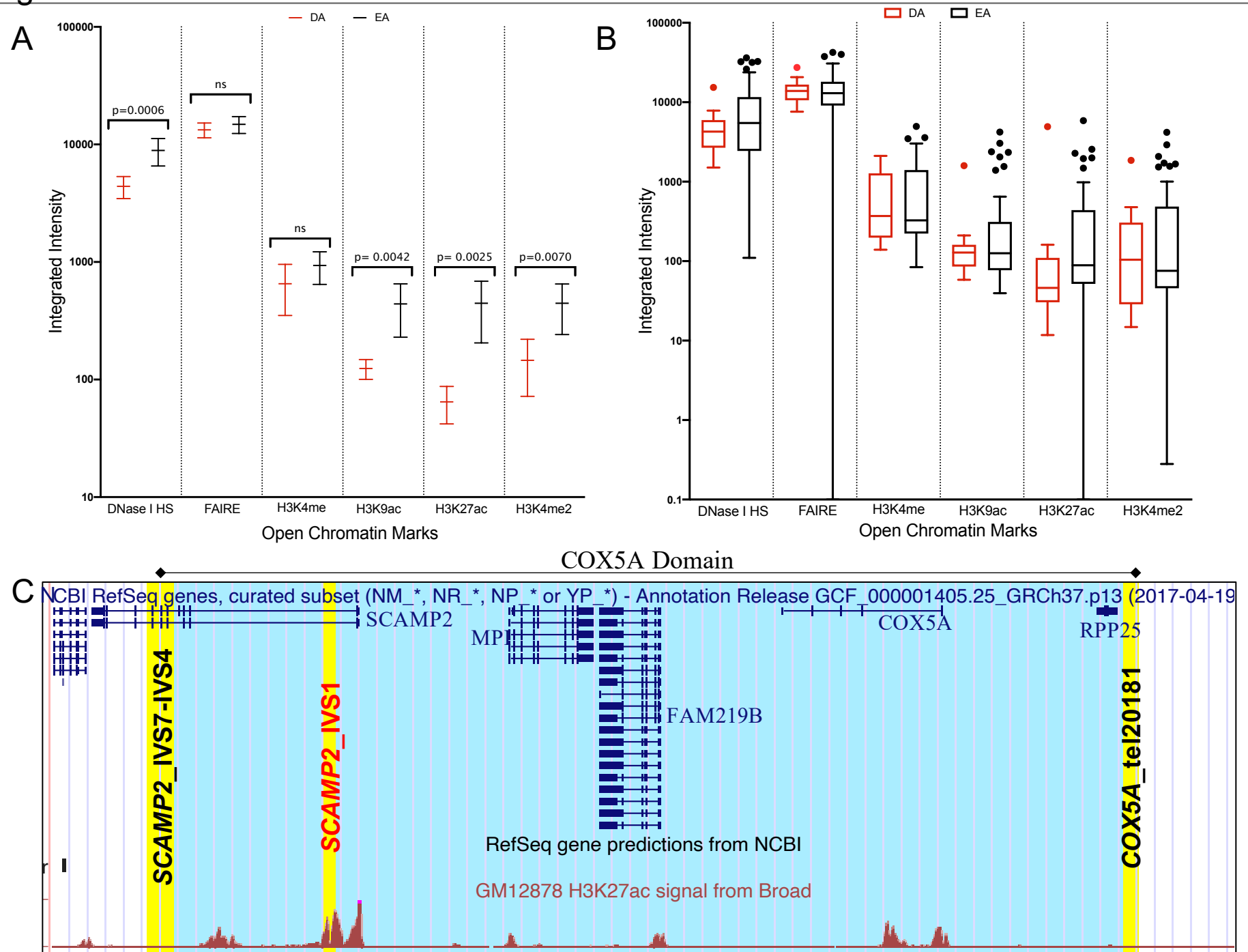

Supplement: Supplementary file 3 — Additional file 3. Figure S1: Open chromatin marks at DA loci have lower mean integrated intensities compared to EA loci. A) Integrated intensity values of DA regions (red) were significantly lower than EA regions (black) of DNase I HS, H3K9ac, H3K27ac, and H3K4me2 using an unpaired t-test with Welch’s correction for unequal variances. No significant difference was found between the mean integrated intensity values of DA and EA regions for FAIRE and H3K4me (p>0.05). The 95% confidence intervals for the DA (n = 17, excluding SCAMP2_IVS1) and previously reported EA intervals [n=59, (14)] are shown. B) Distribution of integrated intensity data for each open chromatin mark in new DA intervals (n=18) and previously reported EA intervals (n=59). Data for each open chromatin mark (x-axis) are presented in a box and whisker plot with the limits of each whisker determined by Tukey. Center line of each box represents the median. Outliers are represented by dots beyond the limits of the whiskers of each box plot. A single outlier from the DA group identified in 5 of 6 open chromatin marks was derived from the same interval, SCAMP2_IVS1. C) Genomic map of COX5A domain demonstrates the difference in enrichment of open chromatin mark H3K27ac at the SCAMP2_IVS1 DA probe outlier, compared to the neighbouring DA probe loci SCAMP2_IVS7-IVS4 and COX5A_tel20181. H3K27ac (burgundy) is enriched at the SCAMP2_IVS1 locus relative to all other DA loci in this study and those previously reported. H3K27ac signal is ChIP-seq data from the GM12878 lymphoblast cell line (Broad Institute). UCSC genome browser annotations are indicated for GRCh37/hg19. RefSeq genes with isoforms are dark blue, sc probes with DA loci within chromosome region 15q24.1 are yellow. Turquoise indicates the domain defined by these probes. [file 13039_2021_567_MOESM3_ESM.pdf]

Figure S2

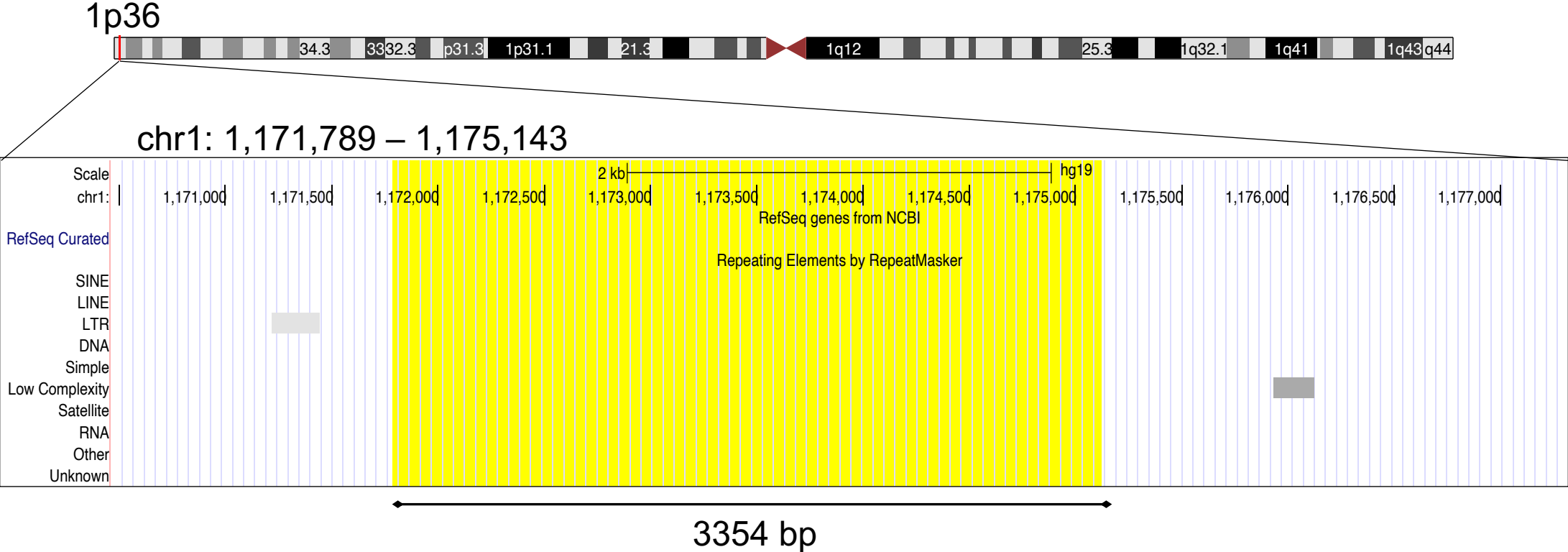

Supplement: Supplementary file 7 — Additional file 7. Figure S2: Genomic map of EA scFISH probe 3.3_1p36. The map expands the genomic EA region of 3.3_1p36 from the chromosome band (red) highlighted on the chromosome ideogram. Created within the UCSC browser (40) using the GRCh37/hg19 human genome assembly, the yellow bar indicates the specific location of the hybridized scFISH probe (chr1:1,171,789-1,175,143). The left margin names each track. Genomic coordinates [hg19] are provided followed by curated RefSeq genes (dark blue when present) and a variety of different repetitive sequences. An intergenic region, no genes are present in this genomic map. The repeating elements (RepeatMasker) are presented in greyscale. Decreasing intensity of grey to white corresponds to increasing divergence between DNA sequences within the same family. ScFISH probes are located within regions that either have no repeating elements or divergent repeating elements (greater than 20% sequence divergence). [file 13039_2021_567_MOESM7_ESM.pdf]
